# Preparation of Porous Hydroxyapatite Using Cetyl Trimethyl Ammonium Bromide as Surfactant for the Removal of Lead Ions from Aquatic Solutions

**DOI:** 10.3390/polym13101617

**Published:** 2021-05-17

**Authors:** Silviu-Adrian Predoi, Carmen Steluta Ciobanu, Mikael Motelica-Heino, Mariana Carmen Chifiriuc, Monica Luminita Badea, Simona Liliana Iconaru

**Affiliations:** 1 Polytech Sorbonne, Sorbonne Universite, 4 Place Jussieu, 75005 Paris, France; silviuadrian00@gmail.com; 2 Lycée Louis-le-Grand, 123 Rue Saint-Jacques, 75005 Paris, France; 3 National Institute of Materials Physics, Atomistilor Street, No. 405A, P.O. Box MG 07, 077125 Magurele, Romania; monibadea78@gmail.com; 4 ISTO, UMR 7327 CNRS Université d’Orléans, 1A rue de la Férollerie, CEDEX 2, 45071 Orléans, France; mikael.motelica@univ-orleans.fr; 5 Life, Environmental and Earth Sciences Division, Research Institute of the University of Bucharest (ICUB), University of Bucharest, 060023 Bucharest, Romania; 6 Microbiology Department, Faculty of Biology, University of Bucharest, 1–3 Portocalelor Lane, 77206 Bucharest, Romania; 7 Academy of Romanian Scientists, Ilfov Street, No. 3, 50044 Bucharest, Romania; 8 Faculty of Horticulture, University of Agronomic Sciences and Veterinary Medicine, 59 Mărăşti Blvd., 11464 Bucharest, Romania

**Keywords:** adsorption, lead, hydroxyapatite, CTAB, removal, ultrasound studies

## Abstract

In the present study, a new low-cost bioceramic nanocomposite based on porous hydroxyapatite (HAp) and cetyl trimethyl ammonium bromide (CTAB) as surfactant was successfully obtained by a simple chemical co-precipitation. The composition and structure of the HAp-CTAB were characterized by X-ray diffraction (XRD), Fourier transform infrared (FTIR) spectroscopy, transmission electron microscopy (TEM), scanning electron microscope (SEM) equipped with an energy dispersive X-ray (EDX) spectrometer, and N_2_ adsorption/desorption analysis. The capacity of HAp-CTAB nanocomposites to remove the lead ions from aqueous solutions was studied by adsorption batch experiments and proved by Langmuir and Freundlich models. The Pb^2+^ removal efficiency of HAp-CTAB biocomposite was also confirmed by non-destructive ultrasound studies. The cytotoxicity assays showed that the HAp-CTAB nanocomposites did not induce any significant morphological changes of HeLa cells after 24 h of incubation or other toxic effects. Taken together, our results suggests that the obtained porous HAp-CTAB powder could be used for the decontamination of water polluted with heavy metals, such as Pb^2+^.

## 1. Introduction

In the recent years, one of the major issues affecting the public health area worldwide is the heavy metals contamination of waters. Heavy metals are non-biodegradable and tend to accumulate in living organisms. On the other hand, the excess of metals in the water stream could inflict severe diseases in people and have a negative impact on the environmental ecosystems [1,2,3,4,5,6,7,8,9,10,11,12,13,14,15,16,17,18]. One of the most current interests in science and public health policy worldwide is the lead carcinogenicity, which is significant for many populations around the globe. Despite the strict regulations on certain uses of lead, there are continuous environmental and occupational sources of exposure in many countries. The lead effects on the hematological system (e.g., inhibition of hemoglobin synthesis and altering of the red blood cells morphology and survival rate, with the occurrence of anemia) have been known and studied for years. [19,20]. In addition, studies regarding the toxic effects of lead against cell membrane components concluded that there is a direct correlation between the toxic effects and lead-induced oxidative damage [19,20,21].

The spreading of largely contaminated zones around the globe and especially the toxic effects of lead ions on human and environmental health have become of great concern. Therefore, great efforts and resources have been made by researchers around the world for finding new methods to resolve the lead pollution problem. One of the most recent studied methods for metals removal from wastewaters is the adsorption of metal ions [3,4,5,6,7,8] using various types of materials. The literature reports numerous studies that showed that apatites (particularly hydroxyapatite, due to its unique structure that confers a strong affinity for various metal ions) could successfully remove metal ions from aqueous solutions [3,9,10].

Recently, surface porogen agents such as chitosan [11], cellulose [12,13], tetraethoxysilane [3], methyltrimethoxysilane [14], etc., have been used for the development of hydroxyapatite-based materials, to increase their adsorption capacity. All these materials have been proposed to be used for aqueous solutions decontamination. Cetyltrimethylammonium bromide (CTAB) was recently proposed as a porogen material used to improve the adsorption capacity of phosphate ceramics. In their study, García et al. [15] reported the use of a new type of microemulsion system based on CTAB/toluene/n-butanol/water for the preparation of hexagonal hydroxyapatite under hydrothermal conditions. The study reported obtaining hydroxyapatite particles with a good population, with typical hexagonal prism-like morphology, with an average particle size around 100 nm. These results are in agreement with other reported studies regarding the obtaining of CTAB-HAp nanocomposites.

Furthermore, Liu et al. [16] also reported that they have successfully developed hydroxyapatite with a needle-like structure in the presence of CTAB, and they have also concluded that in the absence of CTAB and PEG 400, HAp was obtained in the form of particles. In addition, their study concluded that the additions of CTAB, PEG 400, NH_3_OH, and ethanoic acid are crucial for the formation of the nanorods in the nanocomposites. Furthermore, another study conducted by Shih et al. [17], regarding the synthesis of HAp powders, revealed that the addition of CTAB had an important role in the size of the synthesized powders, allowing the obtaining of particle sizes around 20 nm. Nonetheless, until now, just a few studies regarding the utilization of CTAB in the development of composites used for water decontamination have been reported [18]. On the other hand, the utilization of porous phosphate ceramics-based CTAB in water decontamination is not cited in the literature. Therefore, our studies will contribute to fill a knowledge gap regarding the better understanding of the mechanisms and processes that are underlying the adsorption process of metals by porous phosphate ceramics.

The goal of this study was to prepare a new low-cost HAp-CTAB nanocomposite. The use of CTAB as a surfactant has contributed both to the stabilization of HAp and to the creation of more active centers on the surface, thus contributing to the increase of the adsorption capacity of lead ions from contaminated waters. Besides, in this study, the adsorption isotherms and equilibrium of the adsorption processes were considered. Furthermore, the efficacy of HAp-CTAB in removing lead ions from contaminated aqueous solutions has been demonstrated for the first time by non-destructive ultrasound studies.

## 2. Materials and Methods

### 2.1. Materials

Cetyltrimethylammonium bromide ((C_16_H_33_)N-(CH_3_)_3_Br, CTAB), calcium nitrate (Ca(NO_3_)_2_·4H_2_O), ammonium dihydrogen phosphate ((NH_4_)_2_HPO_4_), ammonium hydroxide (NH_3_·H_2_O), and deionized water were purchased from Merck (Merck, Kenilworth, NJ, USA).

### 2.2. Sample Synthesis 

In order to prepare the HAp-CTAB composite (Ca/P molar ratio equal to 1.67), the CTAB and Ca(NO_3_)_2_·4H_2_O was dissolved in 300 mL deionized water resulting Ca-CTAB-containing solution (9 g). The (NH_4_)_2_HPO_4_ was dissolved in 300 mL deionized water resulting P-containing solution. The P-containing solution was added drop by drop into the Ca-CTAB-containing solution and stirred for 6h. The pH value was continually adjusted at 10 during the reaction. The resulting precipitate was washed five times with deionized water. After washing, the precipitate was centrifuged and the HAp-CTAB composite was dried at 100 °C for 24 h.

### 2.3. Characterization of HAp-CTAB Composite

#### 2.3.1. X-ray Diffraction (XRD)

The X-ray diffraction (XRD) pattern of the HAp-CTAB composite was recorded on a Bruker D8 Advance diffractometer with nickel-filtered Cu Kα (λ = 1.5418 Å) radiation (Billerica, MA, USA). The sample was measured at room temperature. The scanning range was 20–60° in 2θ. The incremental step was 0.02° while the time per step was 0.4 s. The average crystal size of the HAp-CTAB composite powder was calculated using the Scherrer equation [22]:

Γ = (0.94 · λ)/(Dcosθ)(1)

where Γ is the full-width at half-maximum (FWHM), λ is the X-ray wavelength, D is the crystal size, and θ is the Bragg angle for the reflection (002).

#### 2.3.2. Fourier Transform Infrared Spectroscopy (FT-IR)

FTIR studies were conducted using a Spectrum BX spectrometer (Perkin Elmer, Waltham, MS, USA). In order to acquire the spectra of analyzed nanoparticles, 1% nanopowder was mixed and ground with 99% KBr. Pellets of 10 mm diameter were prepared by pressing the powder mixture. The spectrum was taken in the range of 400 to 4000 cm^−1^ with a resolution of 4 cm^−1^.

#### 2.3.3. Transmission Electron Microscopy (TEM)

Studies regarding the morphology and particle size of the HAp-CTAB nanocomposites were performed with the aid of a CM 20 (Philips FEI, Eindhoven, The Netherlands) transmission electron microscope (TEM), equipped with a Lab6 instrument.

#### 2.3.4. Scanning Electron Microscopy (SEM)

Furthermore, the morphology of the HAp-CTAB nanocomposites was also investigated using a scanning electron microscopy (SEM) with the aid of a Hitachi S4500 instrument (Hitachi, Tokyo, Japan). In addition, the chemical constituents of the nanocomposites were measured with the aid of energy-dispersive X-ray spectroscopy (EDS).

#### 2.3.5. N_2_ Adsorption/Desorption Analysis

N_2_ adsorption/desorption analysis of HAp-CTAB ceramics were conducted using a Micromeritics ASAP 2020 automatic analyzer at 77K. The sample was degassed at 450 K for 24 h before the measurement. The specific surface area (SSA) was calculated by the Barrett−Emmett−Teller (BET) equation [23]. The adsorption cumulative surface area, the pore size, and volume distribution were calculated based on the Barrett−Joyner−Halenda (BJH) model [24].

### 2.4. Evaluation of the Efficiency of HAp-CTAB Composite in Decontamination

#### 2.4.1. Non-Destructive Ultrasound Studies 

In order to assess the efficiency of HAp-CTAB composite in removing lead from contaminated waters, ultrasonic studies were conducted on the contaminated solutions before and after the removal of lead ions. The signals were obtained using two identical transducers. The frequency of the two transducers was 5MHz and the distance between them was 30 mm. Ninety-one signals were recorded. Three echoes were measured for each recorded signal. To evaluate the efficiency of the HAp-CTAB composite in removing lead from contaminated waters, double distilled water was used as a reference in ultrasonic studies. Ultrasound pulses [25] were used to investigate both lead-contaminated solutions and solutions obtained after lead removal by the use of HAp-CTAB composite.

#### 2.4.2. Batch Adsorption Experiments

In order to assess the capacity of the HAp-CTAB nanoparticles of retaining lead ions from contaminated solutions, batch adsorption experiments were performed. The experiments were conducted at room temperature and in atmospheric conditions using 40 mL silicon tubes filled with lead-contaminated aqueous solutions. The lead concentration in the aqueous solutions varied from 0 to 100 mg/L, and the amount of HAp-CTAB powder was kept at 0.2 g. The pH of contaminated aqueous solutions was constantly adjusted to 5 with the aid of a solution hydrochloric acid (HCl), with a concentration of 0.1 M. For the experiments, the solution volume was kept at 20 mL. A Mixer SRT1 Roller (Stuart Scientific, Staffordshire, UK) was used to shake the silicone tubes containing the contaminated solutions and HAp-CTAB nanocomposites for 24 h in atmospheric condition at a temperature of 25 ± 2 °C. After 24 h, the mixtures were centrifuged at 10,000 rpm, and the supernatant was filtered, recovered, and analyzed by Flame Atomic Absorption Spectrometry (AAS) using a Zeeman HITACHI Z-8100 from Japan Hitachi (Tokyo, Japan) instrument. The AAS investigations were performed in triplicate at a wavelength of 283.3 nm in agreement with the operational conditions for lead.

The adsorption kinetics investigations were evaluated using both Langmuir and Freundlich adsorption models [26,27,28,29,30]. The amount of adsorbed metal ions onto the adsorbent at equilibrium, Q_e_ (mg/g), was calculated according to:

(2) $$Q_{e}=\frac{\left(C_{i}-C_{e}\right)}{m}\cdot V$$

where C_i_ and C_e_ (mg/L) depicts the initial and equilibrium Pb^2+^ concentrations in mg/L, V (L) represents the volume of the solution and m (g) stands for the mass of the HAp-CTAB. Furthermore, the percentage removal of lead ions from aqueous solutions using HAp-CTAB nanoparticles (R%) was calculated from the equation:

(3) $$R\left(\%\right)=\frac{\left(C_{i}-C_{e}\right)}{C_{i}}\cdot 100$$

in which C_i_ and C_e_ are the initial and equilibrium concentrations of the contaminant ions in mg/L.

Information regarding the adsorption process were obtained by determining the Langmuir constants, q_m_ and K_L_ using the graphical representation of the linear Langmuir equation:

(4) $$\frac{C_{e}}{Q_{e}}=\frac{1}{\left(q_{m}\cdot K_{L}\right)}+\frac{C_{e}}{q_{m}}$$

Moreover, with the aid of the determined constants, R_L_, the separation factor constant, known also as the equilibrium parameter, was determined from the equation:

(5) $$R_{L}=\frac{1}{1+K_{L}C_{i}}$$

Additional studies about the adsorption kinetics of lead ions onto HAp-CTAB nanocomposites were performed using the Freundlich isotherm experimental model described by the following equation:

(6) Qe=kf·Ce1n

where Q_e_ represents the amount of lead ions adsorbed at equilibrium in mg/g, Ce is the metal ion concentration at equilibrium expressed in mg/L, and k_f_ [mg/g (mg/L)-1/n] and n are the Freundlich constants. The Freundlich constants, k_f_ and n, representing the adsorption capacity and the adsorption intensity of the adsorbent, were obtained from the graphical representation (lnQ_e_) function of (lnC_e_) of the linear form of the Freundlich equation

(7) $${\mathrm{lnQ}}_{e}={\mathrm{lnk}}_{f}+\frac{1}{n}{\mathrm{lnC}}_{e}$$

### 2.5. Biological Investigation

The in vitro evaluation of the HAp-CTAB cytotoxicity was performed using the MTT (3-4,5-Dimethylthiazol 2,5-diphenyltetrazolium bromide) assay. The MTT assay was performed as previously reported [31,32,33,34]. The cytotoxicity of the HAp-CTAB was performed using HeLa cells. The viability of the HeLa cells was investigated after being treated for 24 h with HAp-CTAB nanoparticles as well as lead-contaminated and decontaminated solutions. HeLa cell viability was quantified by measuring the absorbance of the suspensions at a wavelength of 595 nm using a TECAN spectrophotometer (Tecan GENios, Grödic, Germany). The MTT assays were performed in triplicate and the results were presented as mean ± SD (standard deviation). The morphology of the HeLa cells was also investigated by optical microscopy after 24 h of incubation with the HAp-CTAB nanoparticles as well as contaminated and decontaminated solutions before and after the lead ions removal batch adsorption experiments.

## 3. Results

**Figure 1:**
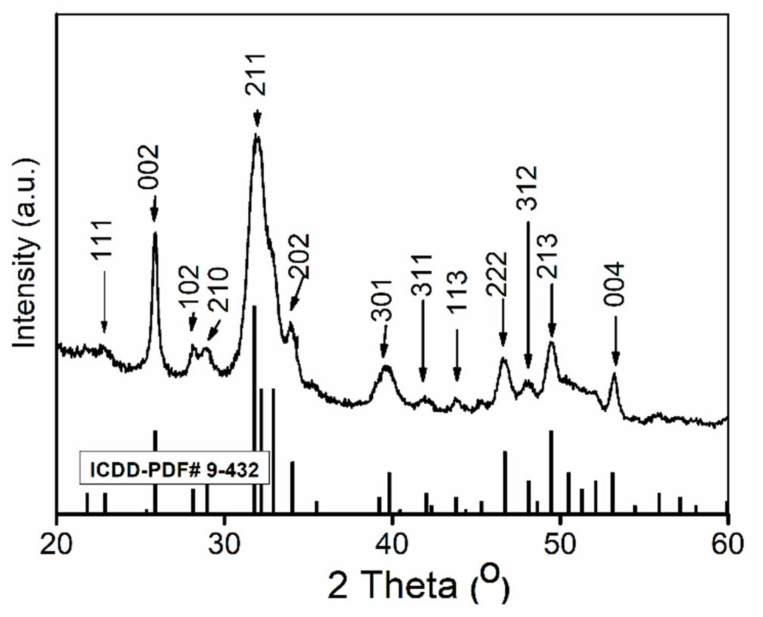
X-Ray Diffraction (XRD) patterns of the HAp-CTAB nanocomposite.

The X-Ray Diffraction (XRD) patterns shown in Figure 1 exhibited that the diffraction peaks of (002), (102), (210), (211), (300), (202), (310), (222), (213), and (004) can be assigned to the standard data (JCPDS No. 9−-432) of single phase hexagonal hydroxyapatite with the space group of P6_3/m_. The XRD patterns shown in Figure 1 exhibited that the diffraction peaks of (002), (102), (210), (211), (300), (202), (310), (222), (213), and (004) can be assigned to the standard data (JCPDS No. 9−432) of single phase hexagonal hydroxyapatite with the space group of P63/m. The calculated values of lattice parameters of HAp-CTAB composite were a = b = 9.43 Å and c = 6.86 Å that were in agreement with the lattice parameters of pure hydroxyapatite a = b =9.4166 Å, c = 6.8745 Å. The value obtained for lattice parameters were in good accordance with the previous studies [35,36]. The average crystallite size of HAp-CTAB powder was 20.5 ± 1 nm. In the XRD patterns showed in Figure 1, no other secondary phase was observed, which highlights the fact that the existence of CTAB did not affect the product structure.

**Figure 2:**
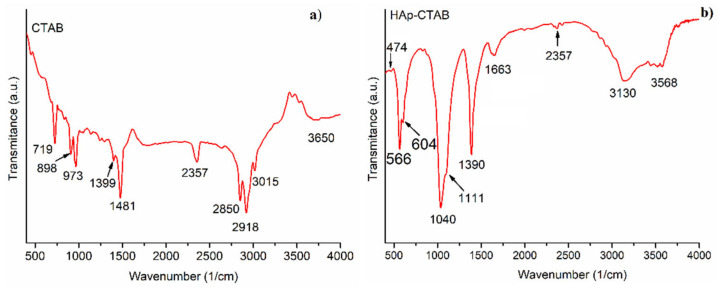
Fourier Transform Infrared Spectroscopy (FT-IR) spectra of CTAB (**a**) and HAp-CTAB composite (**b**).

The Fourier Transform Infrared Spectroscopy (FT-IR) spectra of CTAB and HAp-CTAB were presented ion Figure 2. As can be seen in Figure 2, the bands of 2850 cm^−1^ (ν^a^ (C–H)) and 2918 cm^−1^ (ν^as^(C–H)) were observable only in the CTAB spectrum. On the other side, the spectrum of HAp-CTAB exhibited the vibrations of the PO_4_^3−^ as well as OH^-^ functional groups. The peak at 474 cm^−1^ was assigned to ν_3_ phosphate stretching modes while the peak at 1040 cm^−1^ was attributed to υ_2_ phosphate bending mode. The presence of ν_4_ phosphate bending mode was revealed by the peaks at 566 and 604 cm^−1^. The characteristic peaks at 1399 and 1390 cm^−1^ in the spectra of CTAB and HAp-CTAB were assigned to δ(NH_4_^+^). The peaks at 3015 and 3130 cm^−1^ in the spectra of CTAB and HAp-CTAB were attributed to NH_4_^+^ (υ(NH_4_^-^). The presence in the spectra of peaks at around 1390 cm and in the region 3000–3150 cm^−1^ highlighted the existence of ammonium salt. The existence of adsorbed water in the samples was revealed by the broad bands at around 3650 cm^−1^ in FT-IR spectra of CTAB and 3568 cm^−1^ in FT-IR spectra of HAp-CTAB. The band at 1663 cm^−1^ (FT-IR spectrum of HAp-CTAB) was assigned to the presence of adsorbed water. The peaks associated to the FTIR spectrum of CTAB located in the range of 890–110 cm^−1^ could not be observed in the FTIR spectrum of HAp-CTAB composite due to broad bands present in this region and associated with PO_4_^3−^ vibrations. The achieved results were in accordance with preceding studies [35,37,38,39,40,41] confirming the formation of the HAp-CTAB composite.

**Figure 3:**
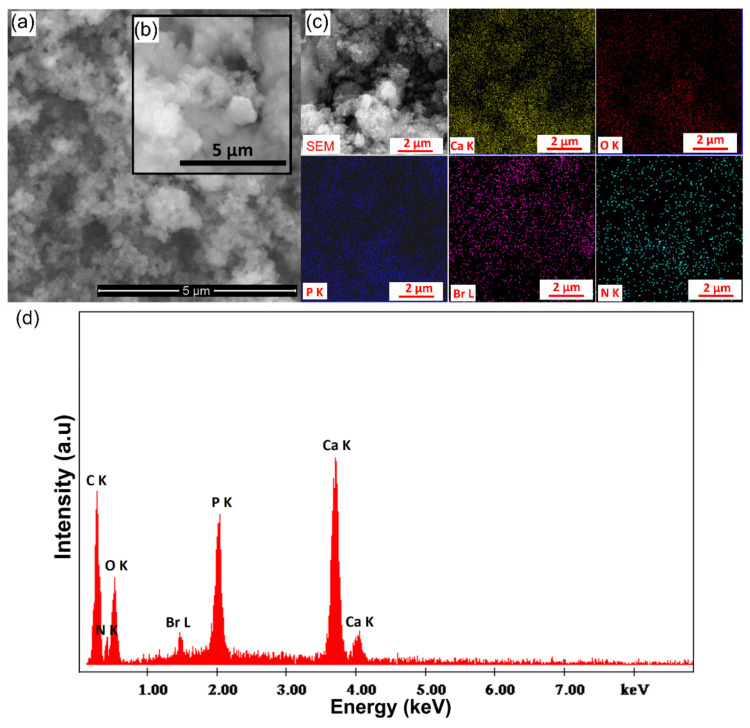
Scanning electron microscopy (SEM) micrograph and zoom area of SEM micrograph (**a**,**b**), SEM micrographs of elemental distribution and elemental distribution maps (**c**), and energy-dispersive X-ray spectroscopy (EDS) spectra (**d**) of HAp-CTAB nanocomposites.

The morphology of the HAp-CTAB nanocomposites has been examined by scanning electron microscopy (SEM). The SEM micrographs and the EDS spectra as well as the elemental distribution map of the HAp-CTAB particles are presented in Figure 3a–d. The SEM images of HAp-CTAB showed that the particles exhibit nanometric dimensions and an almost ellipsoidal morphology (Figure 3a,b). On the other hand, the energy-dispersive X-ray spectroscopy (EDS) spectrum depicted the chemical composition of the HAp-CTAB nanocomposites. In the EDS spectrum, the main chemical constituents of HAp-CTAB, namely calcium (Ca), phosphorus (P), oxygen (O), nitrogen (N), and bromine (Br), were evidenced (Figure 3d). Furthermore, the EDS elemental map showed that the main constituents (Ca, P, O, N and Br) are uniformly distributed in the sample (Figure 3c).

**Figure 4:**
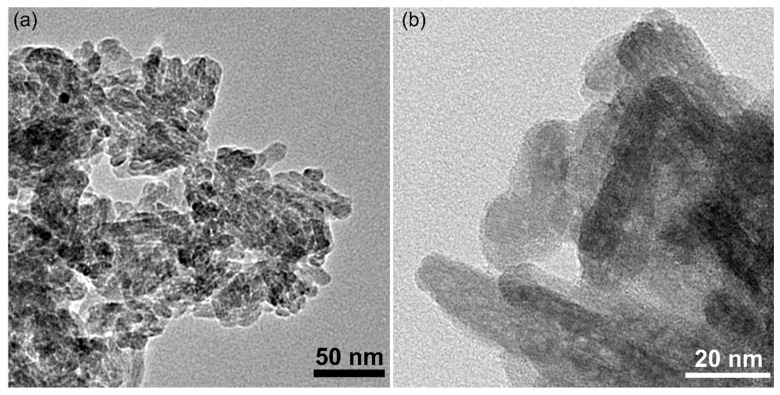
Transmission electron microcopy (TEM) (**a**) and High resolution transmission electron microcopy (HRTEM) (**b**) image of HAp-CTAB nanocomposites.

Furthermore, additional information regarding the morphology of the HAp-CTAB nanocomposites was obtained by transmission electron microcopy (TEM). Figure 4a,b depicts the TEM and HRTEM image of the HAp-CTAB particles. The TEM investigations revealed that the particles have nanometric sizes and present ellipsoidal morphology. Moreover, the HRTEM image emphasized that the nanocomposites exhibit a uniform ellipsoidal morphology with nanometric particle size. The TEM results are in agreement with the SEM findings.

**Figure 5:**
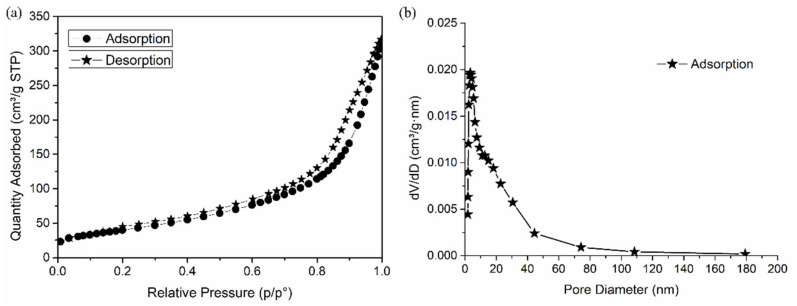
N_2_ adsorption-desorption isotherms (**a**) and pore size distribution graphs (**b**) of HAp-CTAB composite.

Figure 5 presents the specific surface area (SSA) and pore size distribution determined by N_2_ adsorption isotherm. The HAp-CTAB sample exhibits a similar type of IV isotherms according to the IUPAC nomenclature [42]. This behavior is typical for mesoporous materials. Brunauer–Emmett–Teller (BET) SSA of HAp-CTAB composite was 145.8335 m^2^/g with pore volume of 0.4825 cm^3^/g. The BJH adsorption cumulative surface area was found to be 173.1174 m^2^/g. According to previous studies [43,44,45] it was found that the SSA of HAp-CTAB composite was higher that has been previously reported on hydroxyapatite-based materials. In agreement with J. Silvestre-Albero et al. [43], the condensation of the pores indicates that the gas was condensed in a liquid-like phase and this process takes place at a pressure “p” lower than the saturation pressure p_o_ of the liquid in bulk. The extensive and broader hysteresis loop was supported by the Barrett–Joyner–Halenda (BJH) adsorption dv/dw pore volume as exhibited in Figure 5b. During the BJH adsorption, the pore diameter was 12.02 nm.

The capacity of the HAp-CTAB nanocomposites in retaining lead ions from aqueous solutions was studied using flame atomic absorption spectroscopy. The batch adsorption experiments were performed in triplicate at room temperature. The evaluation of the capacity of HAp-CTAB in removing lead ions removal from aqueous solutions was performed by keeping in contact for 24 h, 0.2 g of HAp-CTAB nanocomposites with lead-contaminated solutions. The removal efficiency of Pb^2+^ ions by HAp-CTAB nanocomposites was calculated from the data obtained in the batch adsorption experiments.

**Figure 6:**
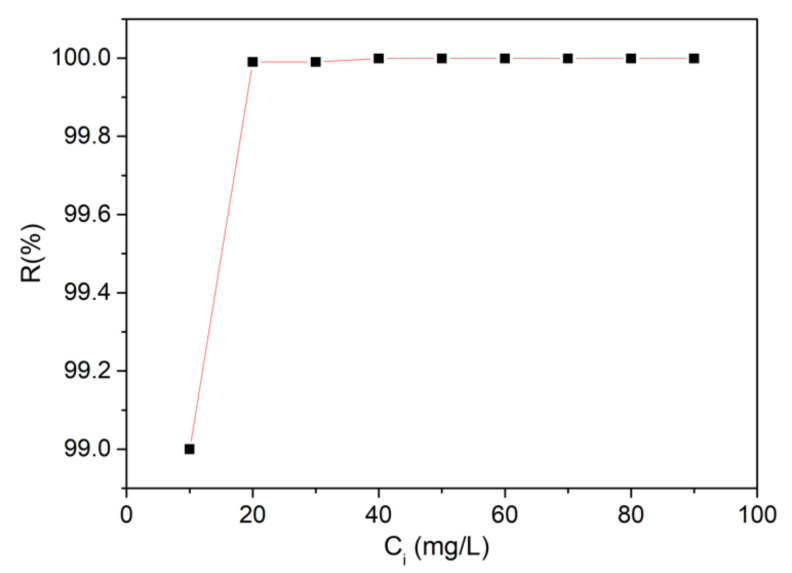
Removal percentage of Pb^2+^ ions from contaminated solutions using HAp-CTAB nanocomposites.

Figure 6 depicts the effect of Pb^2+^ ions concentration from the contaminated solutions on the removal of lead ions from aqueous solutions using 0.2 g of HAp-CTAB nanocomposites. The results have emphasized that the percentage of removal efficiency (R%) is strongly correlated with the initial concentration of Pb^2+^. The data suggested that for a contaminated solution with above 10 mg/L of Pb^2+^, the removal efficiency was higher than 99%. The results highlighted that the HAp-CTAB nanocomposites exhibited a strong affinity for Pb^2+^ ions.

Recently, various decontamination technologies based on the processes of adsorption of metal ions on different materials have been widely proposed in the literature [46,47,48,49,50]. The adsorption processes have often been described by various models such as Langmuir, Temkin, Toth, Freundlich, Hill, etc. [51]. All the models used for describing adsorption processes state that the kinetics plays a very important role in the understanding of the mechanisms involved [52]. The models combine the physico-chemical parameters of the adsorbent and thermodynamic assumptions, thus revealing important information regarding the adsorption mechanisms, surface properties, and also the affinity of the adsorbent [53].

The adsorption process’s kinetics of lead ions by HAp-CTAB nanocomposites were described using both Langmuir and Freundlich adsorption models [26,54].

The data used to compute the isotherms for the lead ions removal using HAp-CTAB nanocomposites were obtained by mixing solutions containing different concentrations of Pb^2+^ with a fix amount (0.2 g) of HAp-CTAB nanocomposites until the reach of the thermodynamic equilibrium. The experiments were performed at ambient temperature (T = 25 ± 2 °C). The obtained data were used to determine the adsorption capacity, which is defined as the amount of metal retained per mass unit. 

**Figure 7:**
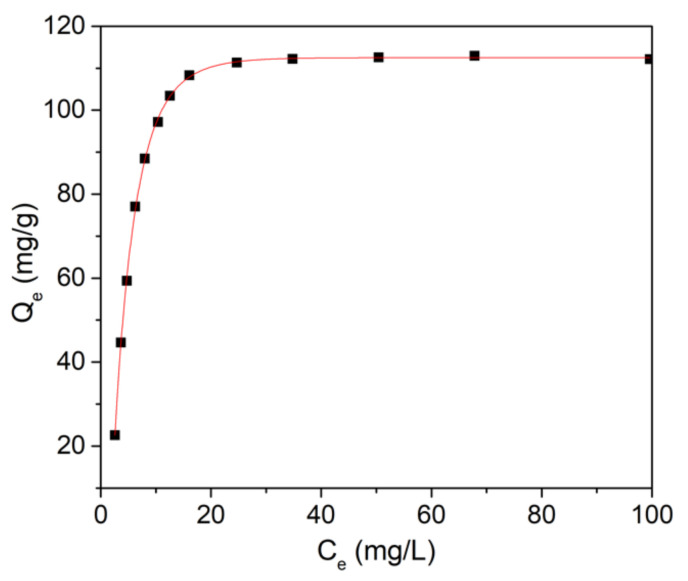
Graphical representation of the amount of material adsorbed at equilibrium by the equilibrium concentration for the adsorption of Pb^2+^ from contaminated aqueous solutions using HAp-CTAB nanocomposites.

The Langmuir model of adsorption which was originally elaborated for the description of the activated carbon gas-solid adsorption phase has also been employed for the quantification of the efficiency of numerous materials used as adsorbents [52]. The empirical model proposed by Langmuir depicts a monolayer adsorption and also describes a process of adsorption that could only happen for a finite number of localized and previously defined areas, which are identical and equivalent [55,56]. The experimental data and the theoretical Langmuir model for the lead ions adsorption from aqueous solutions using HAp-CTAB nanocomposites is presented in Figure 7.

**Figure 8:**
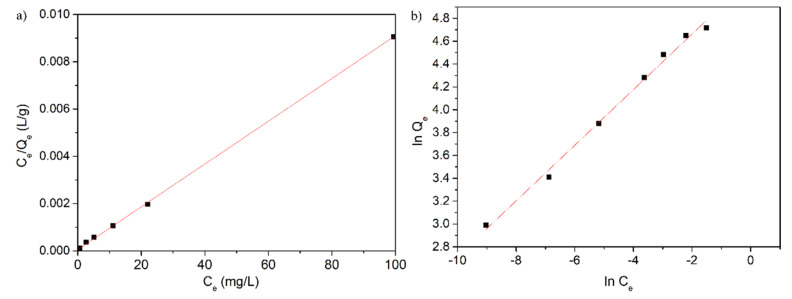
Langmuir (**a**) and Freundlich (**b**) graphical linearized equations for the adsorption of lead ions on HAp-CTAB nanocomposites.

Figure 7 presents the graphical representations of the Pb^2+^ adsorbed ions by HAp-CTAB nanocomposites, on the mass unit (Q_e_) function of the concentration of the Pb^2+^ ions that remained in the contaminated solution after 24 h (C_e_). Furthermore, the Langmuir constants, maximum adsorption capacity (q_m_), and the constant energy associated with the heat of adsorption (K_L_) were determined from the graphical representation of the linear form of the Langmuir equation depicted in Figure 8a.

The data suggested that at ambient temperature, the R^2^ coefficient computed from the Langmuir model isotherm data was equal to 0.999 for HAp-CTAB. These results proved that the Langmuir model fits the data obtained from the batch adsorption experiments well. The results obtained in this study are in concordance with previous reported data on the use of hydroxyapatite based nanocomposites for lead ions adsorption from contaminated aqueous solutions [14,57,58,59,60]. As in previous studies [61,62], the transformation of the nonlinear isothermal Langmuir equation into a linear form using a nonlinear method was performed and the Langmuir constants were calculated.

Recently, numerous studies have been undertaken and their results on the capacity of apatites and hydroxyapatite-based materials to retain metal ions from aqueous solutions were reported in the literature [57,58,59,60,63]. Based on the experimental conditions, the physico-chemical properties of the materials and adsorption parameters, the adsorption capacity of Pb^2+^ ions using hydroxyapatite or hydroxyapatite based materials was reported in the range of 84–620 mg g^−1^ [63]. The results obtained from the batch adsorption experiments data revealed that HAp-CTAB nanocomposites have been extremely successful in removing Pb^2+^ ions from aqueous solutions. The Langmuir constants determined from the data revealed a value of 110.5 mg (Pb)/g for the adsorption capacity and a value of 166.49 L/mg for the K_L_ coefficient. In addition, the Freundlich model was also used in order to better understand the mechanisms involved in the removal of lead ions using HAp-CTAB nanocomposites. The Freundlich adsorption isotherm is often used in the case of a non-ideal and reversible adsorption mechanism. In the Freundlich model, the adsorption is not limited to a monolayer formation and could be applied in multilayer adsorption [63,64,65]. The graphical representations of (lnQ_e_) function of (lnC_e_) for the lead ion adsorption experiments on HAp-CTAB nanocomposites is represented in Figure 8b. The Freundlich constant, k_f_, depicts the adsorption capacity of the materials used as adsorbent, and the 1/n represents the function of the power adsorption from the process [66]. Figure 8b presents the Freundlich linearized equation for the adsorption of lead ions on HAp-CTAB nanocomposites. In the Freundlich model, if the Freundlich constant, n, is equal to 1, then the separation of the two phases is independent of the concentration, while a 1/n value below 1 depicts a normal adsorption process and 1/n value less than 1 indicates a cooperative adsorption process [67].

The values obtained for the Langmuir and Freundich parameters from the adsorption batch experiments are presented in Table 1.

The value from n determined using the linearized form of the Freundlich equation, for the Pb^2+^ ions adsorption experiments on HAp-CTAB nanocomposites, were higher than 1. These results lead to a value of 1/n below 1, which suggested a normal adsorption process. Furthermore, the R_L_ separation factors, which indicates the shape of the isotherm, was also determined using the Freundlich parameters [64]. The R_L_ values equal to 1 depict a linear isotherm, while R_L_ values higher than 1 describe an unfavorable isotherm and a R_L_ value situated between 0 and 1, is associated with a favorable isotherm. A value of R_L_ equal to 0 describes an irreversible isotherm. In the present study, the value of the R_L_ determined from the batch adsorption experiments data was between 0 and 1, depicting a favorable adsorption of Pb^2+^ on HAp-CTAB nanocomposites. Moreover, the data highlighted that the uptake of Pb^2+^ on HAp-CTAB nanocomposites at room temperature and at pH 5 was high, due to the affinity that is relatively large between Pb^2+^ and HAp-CTAB nanocomposites. Moreover, taking into consideration the correlation coefficients (R^2^) determined from fitting the experimental data using both Langmuir and Freundlich theoretical models, it was observed that the experimental equilibrium data of Pb^2+^ sorption onto HAp-CTAB nanocomposites was best described using the Langmuir model. The results of the batch experiments and the data obtained using Langmuir and Freundlich models suggested that HAP-CTAB nanocomposites presented a strong affinity for the adsorption of Pb^2+^ ions from aqueous solutions, therefore rendering HAp-CTAB nanocomposites good candidates in the development of new technologies for water treatment.

An ultrasonic characterization was performed on both polluted and depolluted samples in order to acquire information about the efficiency of removing lead ions from contaminated waters. This method of analysis is non-invasive and can provide information about the solid particles in the analyzed liquid. Since water is considered to be the most stable fluid, we chose double-distilled water as a reference fluid because of its high purity. Ultrasonic measurements revealed properties of the samples by studying the behavior of the spectral amplitudes. Their attenuation in time provided important information about the stability of the analyzed fluid. The effect of HAp-CTAB in removing lead ions from contaminated water is presented below using double-distilled water as a reference for all measured and analyzed signals.

**Figure 9:**
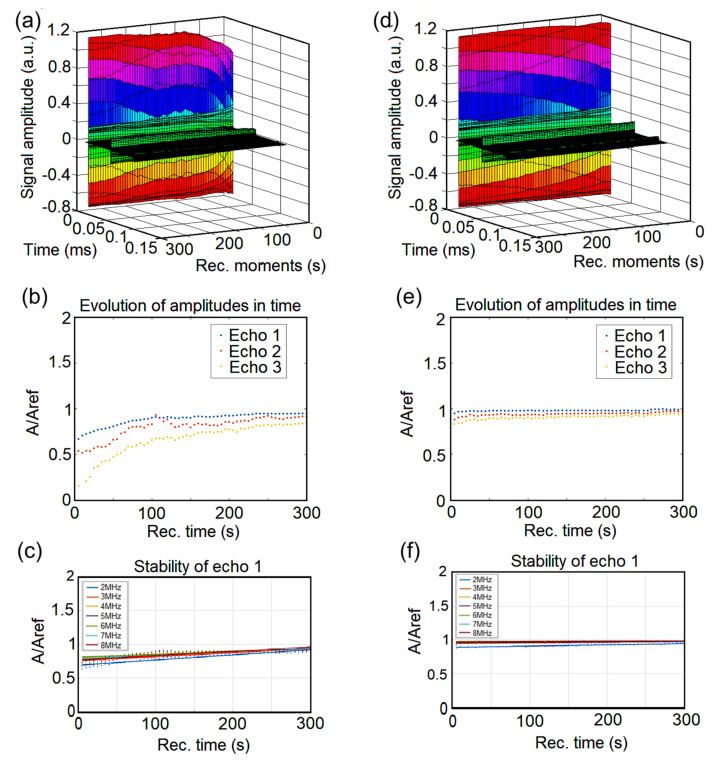
Acquired signals before (**a**) and after (**d**) lead removal. Evolution of amplitude in time before (**b**) and after lead removal (**e**); stability of echo 1 before (**c**) and after (**f**) lead removal.

Figure 9a,d reveals the amplitude of the signals obtained at each moment in the interval 0–300 s, before and after the removal of the lead from the contaminated water. The signals were registered at 5 s intervals for lead-contaminated water (a) and decontaminated water (d). The constant peak amplitudes of the first echo is presented in color. The second and third echoes were significantly weaker and are shown in black.

(Figure 9b,e) illustrates how the relative spectral amplitudes (RSA) of the three desired echoes change with time. The reference used for the amplitudes is water (A_ref_). For the sample representing lead-contaminated water, the relative amplitude (A/A_ref_) of the three echoes is much lower than 1 even at Rec.time = 300s (Figure 9b). After decontamination (Figure 9e), it is observed that all three analyzed echoes have (A/A_ref_) ∈ [0.8; 1] for Rec. Time (s)∈ [0; 40]. Also, from Figure 9e, there is (A/A_ref_) ∈ [0.9; 1] for Rec.Time (s) ∈ [40; 150]. Furthermore, for Rec. Time (s) ≫ 150, (A/A_ref_) ≈ 1. It is noticed (Figure 9e) that after decontamination, for the first echo, (A/A_ref_) varies slowly from 0.98 to 1 for Rec.Time (s) ∈ [50; 250]. Considering the fact that (A/A_ref_) = 1 for the signal through double-distilled water, it can be said that the biocomposite HAp-CTAB has a very good efficiency in removing lead from the contaminated water.

As it could be observed, the presence of the pollutant renders the sample unstable. In order to track the stability of the samples, Figure 9c,f shows how the RSA of echo 1 varies as function of time, over several frequencies, before/after decontamination. For the contaminated water (Figure 9c), a divergence was noticed for all frequencies compared to the double distilled water (whose equilibrium value has been taken as reference). The values of the RSA for echo 1 in decontaminated water were constant at 1 (Figure 9f). This result showed that the analyzed water after decontamination has the same behavior to that of double distilled water. Consequently, it can be said that this study certifies the effectiveness of HAp-CTAB in lead removal from water.

**Figure 10:**
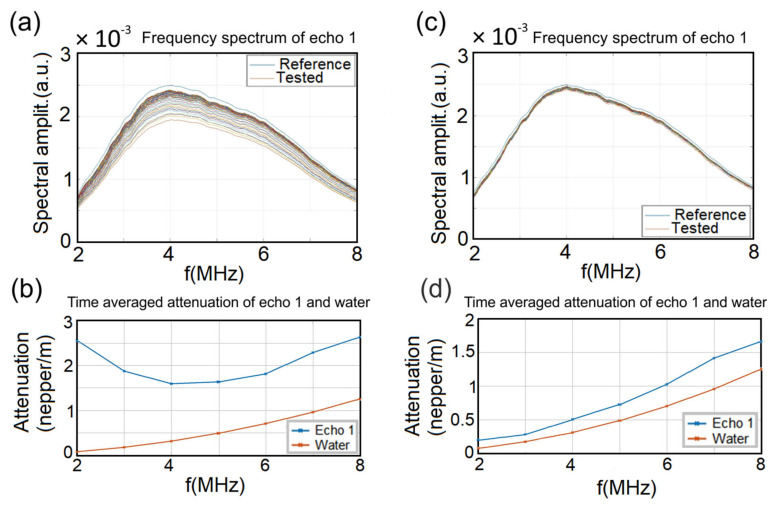
Frequency spectrum of echo 1 before (**a**) and after lead removal (**c**). Time averaged attenuation of echo 1 and water before (**b**) and after lead removal (**d**).

It has been observed by signal analysis that echo 1 has the highest accuracy, due to its direct path between the transducers. For this reason, the rest of the analysis concerns only echo 1 in all recorded signals (Figure 10a–c). The behavior of the spectral amplitudes for echo 1 (Figure 10a) in the case of polluted water indicates a visible convergence in time towards the reference across all spectra, from lower to higher amplitudes. On the other hand, it can be observed that the spectral amplitude of the decontaminated water is very close to that of the reference (Figure 10c).

The efficiency of the HAp-CTAB biocomposite in removing lead ions from contaminated water was also highlighted in Figure 10b, which shows the time-averaged evolution of the spectral attenuation of echo 1. The maximum attenuation at 2 MHz in lead-polluted water was 2.56 Np/m (Figure 10b). After removing the lead, the maximum attenuation at 2 MHz was equal to 0.24 Np/m, a value very close to that of the double distilled water (Figure 10d). Moreover, in the case of polluted water, a minimum attenuation at 4 MHz was observed. On the other hand, the time-averaged evolution of the spectral attenuation of echo 1 for the decontaminated water sample followed that of the reference water (Figure 10d).

Non-destructive ultrasound studies could provide important information about solutions and suspensions because the ultrasound velocity through solutions and suspensions in the linear approximation of small perturbations depends on the average density and average compressibility [68,69,70]. The study on “removal of zinc ions using hydroxyapatite and study of ultrasound behavior of aqueous media” presented the experimental results of ultrasound measurements on water, contaminated water, and water after decontamination by temporal signal analysis allows determining the time differences between equivalent echoes in different fluids, with an accuracy of 1 ns [70]. In a recent study on “removal and oxidation of As (III) from water using iron oxide coated CTAB as adsorbent”, the efficiency of the biocomposite in the elimination of arsenic ions was evaluated by non-destructive ultrasound studies based on the time-mediated evolution of spectral attenuation of echo 1 and double distilled water [71]. The stability parameter calculated for the first echo clearly highlighted the efficiency of the biocomposite in removing arsenic from polluted water [71].

**Figure 11:**
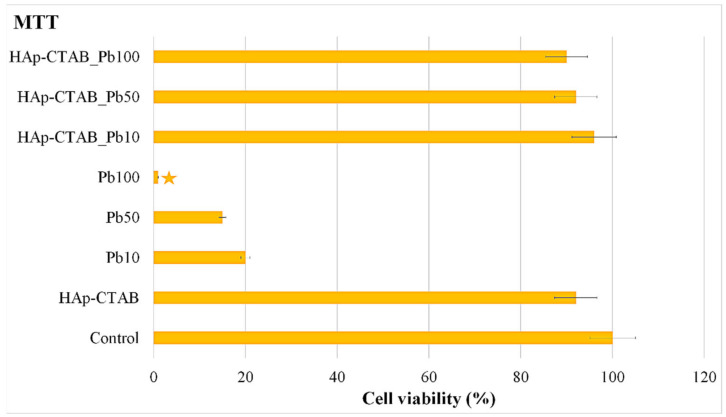
Cell viability of HeLa cells incubated with water in the presence of HAp-CTAB nanocomposites, Pb^2+^ contaminated solutions, and decontaminated solutions using HAp-CTAB nanocomposites. HeLa cell culture was used as the control.

Furthermore, the cytotoxicity of the HAp-CTAB nanocomposites was assessed by performing in vitro cytotoxicity studies on HAp-CTAB nanocomposites, solutions contaminated with various concentrations of Pb^2+^ ions, and decontaminated aqueous solutions. For this purpose, the in vitro cell viability of HeLa cell incubated for 24 h with HAp-CTAB nanocomposites, lead-contaminated solutions, and decontaminated solutions using HAp-CTAB nanocomposites was investigated using the MTT assay. The results of the MTT assay, regarding the HeLa cell viability after treatment with solutions contaminated with lead ions at different concentrations (10 mg/L (Pb10), 50 mg/L (Pb50), and 100 mg/L (Pb100)) and also with the decontaminated solutions using HAp-CTAB nanocomposites (HAp-CTAB_Pb10, HAp-CTAB_Pb50, and HAp-CTAB_Pb100) are presented in Figure 11. The results of the MTT assay highlighted that the solutions contaminated with lead ions exhibited a higher toxicity towards HeLa cells. In addition, the data suggested that the HeLa cell viability has been greatly influenced by the lead ions concentration. The MTT results showed that the cell viability of the HeLa cells decreased considerably with the increase of the lead ions concentrations from 20% for the solutions containing 20 mg/L to 1% for the solutions containing 100 mg/L lead ions. The HeLa culture that has not been treated with any sample was used as control, and all the other values were normalized according to it. Lead is a non-biodegradable naturally occurring element that has been reported to have a high degree of toxicity [72,73,74]. Studies have reported that even small doses of lead could produce devastating effects on various organ systems such as the nervous system, the red blood cells, hematopoietic system, endocrine system, reproductive system, and the kidneys, which are considered to be the primary targets of lead toxicity [72,73,74].

More than that, the results of the MTT assays conducted on the aqueous decontaminated solutions using HAp-CTAB nanocomposites are also presented in Figure 11. The data showed that the decontaminated solutions did not exhibit a noticeable effect on the cell viability of the HeLa culture after 24 h of incubation. The MTT assay results highlighted that for the decontaminated solutions, the cell viability of the HeLa culture was above 90%. These results suggested that the HAp-CTAB nanocomposites could be used for the removal of lead ions from contaminated solutions, without inducing cytotoxic effects on the living organisms. Moreover, the cytotoxicity of the HAp-CTAB nanocomposites was also studied, and the results of the MTT assay are depicted in Figure 11. The data revealed that HAp-CTAB nanocomposites exhibited no toxicity on HeLa cells after 24 h of incubation.

**Figure 12:**
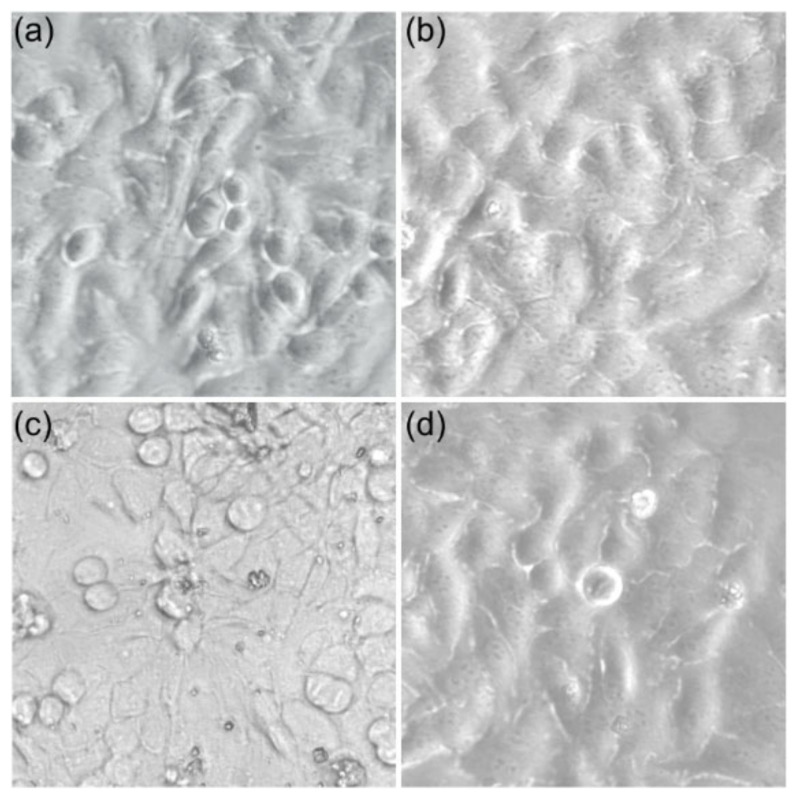
The morphology of the HeLa cells used as control (**a**); incubated with HAp-CTAB nanocomposites in water (**b**), incubated with Pb^2+^ contaminated solutions (**c**), and incubated with solutions decontaminated using HAp-CTAB nanocomposites (**d**).

Complementary information regarding the toxicity of the HAp-CTAB nanocomposites and also of the lead-contaminated and decontaminated solutions was obtained by visualizing the HeLa cells after 24 h of incubation with the aid of an optical microscope. The morphology of the HeLa cells incubated with lead-contaminated solutions at a concentration of 50 mg/L, decontaminated solutions using HAp-CTAB nanocomposites, and HAp-CTAB nanocomposites are depicted in Figure 12.

The visualization of the HeLa cells after 24 h of incubation with the samples confirmed the results from the quantitative MTT assay and suggested that HAp-CTAB nanocomposites did not induced any noticeable morphological changes of the HeLa cells after 24 h of incubation. The morphology of HeLa cells was also not modified after being incubated with the decontaminated solutions. On the other hand, the results of the optical microscopy visualization emphasized that the morphology of the HeLa cells incubated with the lead-contaminated aqueous solution was significantly altered indicating that the lead ions exhibited a strong toxic effect on the HeLa cells morphology. The results are in good agreement with the data obtained from the MTT cytotoxicity assay and with previously reported data regarding the lead toxicity on different cell types [19,75,76,77,78]. The preliminary findings regarding the toxicity of the HAp-CTAB combined with their removal efficiency demonstrated that they could be suitable in the development of novel environmental remediation technologies, which are of great interest worldwide.

Our preliminary results highlight that HAp-CTAB nanocomposites exhibited a good affinity towards lead ions without exhibiting toxicity against HeLa cells, thus being promising leads for the successful development of new water remediation technologies.

## 4. Conclusions

The present study was aimed to obtain, for the first time, a porous bioceramic composite based on hydroxyapatite (HAp) with cetyl trimethyl ammonium bromide (CTAB) as a surfactant, using the co-precipitation method. The HAp-CTAB biocomposite was used as an effective adsorbent in order to remove the lead ions from contaminated aqueous solution. The calculated values of lattice parameters of HAp-CTAB composite were in agreement with the lattice parameters of pure hydroxyapatite. The FTIR studies confirmed the formation of the HAp-CTAB composite. The porosity of HAp-CTAB has been demonstrated by N_2_ adsorption/desorption analysis.

Furthermore, the HAp-CTAB nanocomposites’ capacity of lead adsorption from aqueous solutions was studied by batch adsorption experiments. The adsorption of lead ions followed the Langmuir adsorption isotherm model, suggesting that the HAp-CTAB nanocomposites presented a high affinity towards lead ions and have successfully removed them from the contaminated aqueous solutions. The non-destructive ultrasound studies confirmed the effectiveness of HAp-CTAB in removing lead ions from an aqueous solution. In addition, the HAp-CTAB nanocomposites proved to be not cytotoxic, while the lead-contaminated solutions presented a high toxicity against HeLa cells, the toxicity degree being strongly correlated with the lead ions concentration.

## Figures and Tables

**Table 1:** Langmuir and Freundlich isotherm parameters for Pb^2+^ adsorption onto HAp-CTAB nanocomposites.

Sample	Langmuir			Freundlich		
**HAp-CTAB**	**R^2^**	**q_m_ (mg/g)**	**K_L_** **(L/mg)**	**R^2^**	**n**	**k_f_**
	0.999	110.5	166.49	0.993	4.11	172.25

## Data Availability

Data is available on demand from the corresponding author.
